# Sensing Techniques in Virtual Reality for Human Interaction: A Bibliometric Analysis

**DOI:** 10.3390/s26113556

**Published:** 2026-06-03

**Authors:** Antonio del Bosque, Pablo Fernández-Arias, Diego Vergara

**Affiliations:** Technology, Instruction and Design in Ençgineering and Education Research Group (TiDEE.rg), Facultad de Ciencias y Artes, Universidad Católica de Ávila (UCAV), Calle Canteros s/n, 05005 Ávila, Spain; antonio.bosque@ucavila.es (A.d.B.); pablo.fernandezarias@ucavila.es (P.F.-A.)

**Keywords:** virtual reality, sensing technologies, human–computer interaction, wearable sensors, haptic feedback, physiological sensing, multimodal sensing

## Abstract

**Highlights:**

**What are the main findings?**

- VR sensing research is converging toward human-centered, multimodal interaction paradigms beyond purely visual immersion.
- The field is structurally consolidated yet conceptually heterogeneous, with haptic and tactile systems appearing comparatively less represented within the thematic structures.

**What are the implications of the main findings?**

- Future VR research is increasingly associated with wearable, physiological, and intelligent sensing approaches.
- Haptic feedback and sensor fusion emerge as key technological bottlenecks and major opportunities for innovation.

**Abstract:**

Virtual reality (VR) has emerged as a key technology for immersive human–computer interaction, where sensing systems are essential for enabling natural, adaptive, and multisensory experiences. However, the scientific landscape of sensing techniques in VR remains fragmented across disciplines, lacking a comprehensive and integrative perspective. In this study, a bibliometric and science mapping analysis was conducted to systematically evaluate research trends, structures, and developments in sensing technologies for VR-based human interaction. A dataset of 2259 peer-reviewed articles (2005–2025) retrieved from Scopus and Web of Science was analyzed. The results indicate a steady growth in scientific production (5.37% annual growth rate) and a highly collaborative research environment, structured around a limited core of journals and dominated by leading countries such as China (18.0%) and the United States (17.8%). Conceptual and thematic analyses reveal a transition toward human-centered and interaction-driven approaches, with increasing emphasis on multimodal, wearable, and physiological sensing technologies. At the same time, areas such as haptic and tactile feedback appear comparatively less represented within the analyzed thematic structures. The analyzed bibliometric trends indicate increasing thematic convergence between sensing technologies, materials science, and intelligent systems within VR research, with growing research interest in integrated and multimodal sensing approaches.

## 1. Introduction

Virtual Reality (VR) has rapidly evolved from a niche visualization technology into a cornerstone of immersive digital interaction, enabling users to engage with computer-generated environments in a highly realistic and interactive manner [1,2]. By combining visual, auditory, and increasingly haptic and physiological feedback, VR systems generate a strong sense of presence and embodiment, positioning them as a key component in next-generation human–computer interaction (HCI) paradigms [3].

The growing maturity of VR technologies has driven their widespread adoption across diverse domains. In engineering, VR is extensively used for design optimization, digital twins, and simulation-based training in complex or hazardous scenarios [4,5]. In academia, immersive environments facilitate experiential learning and enhance student engagement beyond traditional pedagogical approaches [6,7]. In healthcare, VR applications have expanded into rehabilitation, surgical planning, mental health therapies, and neurocognitive assessment, demonstrating measurable benefits in both clinical and research settings [8,9]. This multidisciplinary expansion underlines the strategic importance of VR as a technological enabler with significant societal and industrial impact.

Nonetheless, the effectiveness of VR systems is fundamentally constrained by their ability to capture, interpret, and respond to human actions and physiological states in real time [10,11]. In this context, sensing technologies emerge as a critical bottleneck. While advances in graphics rendering and computational power have significantly improved visual immersion, the fidelity of interaction remains highly dependent on the performance, accuracy, and integration of sensing systems [12]. Consequently, the development and optimization of sensors for VR applications constitutes a central challenge, directly determining user experience quality, interaction naturalness, and system adaptability [13].

Among the wide range of sensing technologies employed in VR, mechanical sensors based on electromechanical transduction mechanisms play a dominant role in enabling human interaction. Here, three types of sensors are widely used: (i) piezoresistive sensors, which transduce mechanical deformation into variations in electrical resistance, are widely utilized in wearable systems for motion tracking, strain sensing, and gesture recognition due to their simplicity, scalability, and compatibility with flexible substrates [14]; (ii) piezoelectric sensors generate electrical signals in response to dynamic mechanical stress, making them particularly suitable for detecting pressure, vibration, and impact, as well as for integration into haptic feedback systems where real-time responsiveness is essential [15,16]; and (iii) capacitive sensors represent another key technology, enabling high-sensitivity detection of touch, proximity, and gesture-based interactions with relatively low power consumption [17,18]. These sensors are broadly integrated into human–machine interfaces and are particularly relevant in lightweight and portable VR systems.

Moreover, there are other sensors that are employed for VR purposes. On the one hand, optical sensing technologies, including infrared tracking, structured light systems, and camera-based motion capture, constitute the backbone of spatial tracking in many commercial VR platforms, providing accurate position and orientation data for head, hand, and full-body tracking [19]. On the other hand, inertial sensing systems based on inertial measurement units (IMUs), which combine accelerometers, gyroscopes, and magnetometers, are extensively used to capture dynamic motion and orientation [20]. Their integration with other sensing modalities enables sensor fusion approaches that improve robustness and accuracy in complex interaction scenarios.

Beyond mechanical and kinematic sensing, there is a growing trend toward the incorporation of physiological sensors in VR systems. Technologies such as electromyography (EMG), electroencephalography (EEG), electrodermal activity (EDA), and heart rate monitoring enable the acquisition of bio-signals that reflect users’ cognitive, emotional, and physical states [21,22]. This shift toward bio-integrated sensing opens new possibilities for adaptive and personalized VR experiences, particularly in applications related to healthcare, training, and affective computing.

Furthermore, recent advances in materials science are driving the development of next-generation sensors based on flexible, stretchable, and nanostructured materials, including carbon nanotubes, graphene, and hybrid nanocomposites [23,24]. These materials enable the fabrication of highly sensitive, lightweight, and conformable sensors that can be seamlessly integrated into wearable devices, significantly enhancing the quality and naturalness of interaction in immersive environments. Despite these promising developments, challenges related to stability, hysteresis, scalability, and long-term reliability remain largely unresolved [25,26].

**Figure 1:**
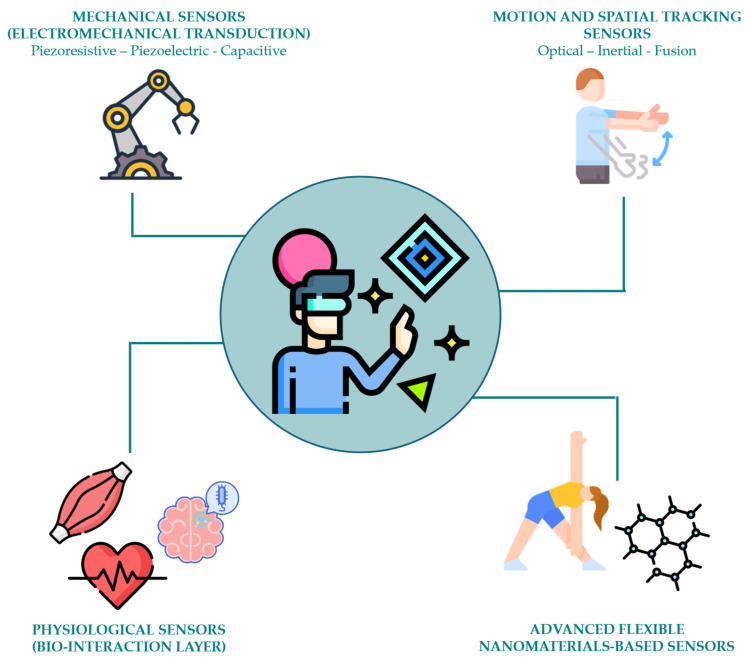
Classification of sensing technologies in VR for human interaction.

A comprehensive classification of sensing technologies used in VR for human interaction is illustrated in Figure 1, highlighting the integration of mechanical, kinematic, physiological, and advanced material-based sensing approaches within immersive environments. In the context of this study, the concept of human interaction refers to sensing technologies associated with interaction input systems, physiological monitoring, sensory perception, wearable interfaces, immersive interaction, and other human-centered VR applications related to user experience and multisensory integration.

Despite the rapid expansion of VR technologies and the critical role of sensing systems, the existing literature remains highly fragmented across disciplines such as materials science, electronics, computer science, neuroscience, and biomedical engineering. Most studies focus on specific sensor technologies or application domains, lacking a comprehensive and integrative perspective of the field. There is a notable absence of analyses that map the evolution, structure, and emerging trends of sensing techniques in VR for human interaction. To address this gap, this study presents a comprehensive bibliometric and science mapping analysis of sensing techniques in VR, with a specific focus on their role in enabling human interaction. By leveraging advanced bibliometric tools and methodologies, this work aims to identify key research trends, influential publications, leading authors and institutions, and emerging technological directions. Finally, this analysis provides a structured and quantitative overview of the field, supporting future research and development in next-generation VR sensing systems.

## 2. Experimental Procedure

### 2.1. Bibliometric Framework and Study Design

This study adopts a structured bibliometric methodology to systematically analyze the scientific landscape of sensing techniques in VR for human interaction. Bibliometric analysis is particularly suitable for evaluating research evolution in multidisciplinary fields that integrate engineering, materials science, computer science, neuroscience, and human–computer interaction. This approach enables the quantitative assessment of publication trends, intellectual structure, collaboration networks, and thematic development, providing a comprehensive understanding of the field.

**Figure 2:**
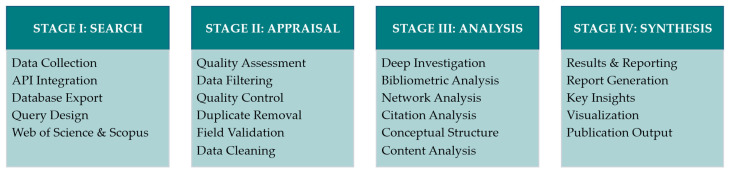
SAAS workflow applied in the bibliometric analysis, including the four main stages: search, appraisal, analysis, and synthesis.

The methodological framework followed a four-stage workflow based on the SAAS model (Search, Appraisal, Analysis, and Synthesis), implemented through the Bibliometrix (version 5.3.0) in R (version 4.5.1) package and its Biblioshiny interface [27,28]. This workflow ensures methodological rigor, transparency, and reproducibility throughout the bibliometric process. As illustrated in Figure 2, the workflow is structured into four main stages: (i) search, systematic collection of bibliographic data from major academic databases through structured query design and database export procedures; (ii) appraisal, quality assessment and filtering of the collected data, including duplicate removal, metadata validation, and data cleaning to ensure dataset consistency; (iii) analysis, application of advanced bibliometric and science mapping techniques, such as citation analysis, network analysis, and conceptual structure identification; and (iv) synthesis, integration of results into meaningful insights through visualization, reporting, and scientific interpretation, leading to the final research output.

Here, it is important to note that this workflow is iterative rather than strictly linear. Insights obtained during the analysis and synthesis stages may require refinement of earlier phases, such as adjusting the search strategy or filtering criteria. This iterative cycle enhances the robustness, reliability, and coherence of the bibliometric analysis.

### 2.2. Data Retrieval and Sources

The bibliographic dataset was compiled from two major scientific databases: Scopus and Web of Science Core Collection (WoS). These platforms were selected due to their comprehensive coverage of peer-reviewed literature, standardized indexing systems, and robust citation tracking capabilities. The combination of both databases ensures broad coverage of publications related to VR sensing technologies while minimizing database-specific bias. All records were exported with full bibliographic metadata, including authors, affiliations, titles, abstracts, keywords, and citations, in formats compatible with bibliometric analysis tools.

### 2.3. Query Design and Conceptual Structure

The search strategy was developed through a structured and reproducible approach aimed at capturing the multidisciplinary nature of the field. Given the convergence of fields such as engineering, computer science, neuroscience, and materials science, a comprehensive query design was required to ensure both high recall and thematic specificity.

**Figure 3:**
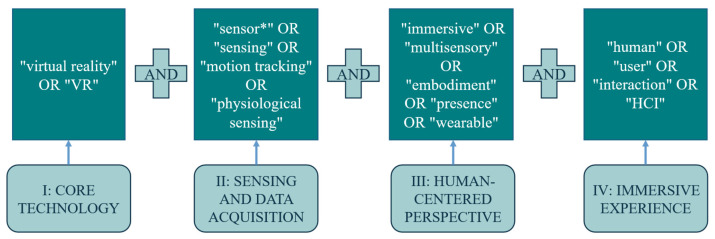
Conceptual framework used to construct the bibliographic search strategy based on four thematic axes: VR technology, sensing, human interaction, and immersive experience. For Scopus, the query used is (TITLE-ABS-KEY (“virtual reality” OR “VR”) AND TITLE-ABS-KEY (“sensor*” OR “sensing” OR “motion tracking” OR “physiological sensing”) AND TITLE-ABS-KEY (“human” OR “user” OR “interaction” OR “HCI”) AND TITLE-ABS-KEY (“immersive” OR “multisensory” OR “embodiment” OR “presence” OR “wearable”)) AND PUBYEAR > 2004 AND PUBYEAR < 2026. For WoS TS = ((“virtual reality” OR “VR”) AND (“sensor*” OR “sensing” OR “motion tracking” OR “physiological sensing”) AND (“human” OR “user” OR “interaction” OR “HCI”) AND (“immersive” OR “multisensory” OR “embodiment” OR “presence” OR “wearable”)) AND PY = (2005–2025). Database search performed in April 2026. Wildcard (*) is used to term variation.

To achieve this, the search query indicated in Figure 3 was constructed based on a conceptual framework composed of four complementary thematic axes: (i) core VR technology, (ii) sensing and data acquisition, (iii) human-centered perspective, and (iv) immersive experience. Each axis was designed to represent a key dimension of the research topic, ensuring that the retrieved publications addressed not only VR as a technological platform but also its interaction mechanisms and user-centered implications.

Within each axis, relevant keywords and synonymous expressions were identified through an iterative process involving preliminary literature exploration and expert-driven refinement. These terms were combined using the Boolean operator OR to capture lexical variability and ensure broad coverage. Subsequently, the four thematic axes were interconnected using the Boolean operator AND, enforcing that retrieved documents simultaneously addressed all core dimensions of the study. The use of wildcard truncation (*) allowed the inclusion of multiple word variations, thereby increasing the sensitivity of the search. At the same time, the combination of axes ensured that irrelevant studies, such as those focused solely on VR hardware without sensing components or purely theoretical HCI works, were minimized.

This query design was iteratively refined through pilot searches, during which retrieved records were manually inspected to assess relevance and adjust keyword combinations accordingly. During this process, retrieved records were manually examined to evaluate thematic relevance, identify potential false positives, and optimize keyword combinations and Boolean structures accordingly. To further strengthen methodological consistency and minimize selection bias, the screening process was subsequently conducted by the three authors following a multi-step evaluation protocol. Prior to the complete screening stage, a calibration exercise was carried out using a representative subset of publications to standardize the interpretation of the inclusion and exclusion criteria. The calibration subset consisted of 50 randomly selected records from the preliminary dataset, which were independently screened by the three authors based on titles, abstracts, keywords, and bibliographic metadata. Publications explicitly related to sensing technologies in VR for human interaction (including wearable sensing, physiological monitoring, motion tracking, immersive interaction, and multisensory feedback) were classified as included, whereas publications unrelated to immersive VR interaction, lacking sensing-related content, or focused exclusively on non-human-centered VR applications were excluded. Records presenting ambiguity during the initial classification stage were temporarily categorized as requiring further evaluation and subsequently discussed jointly until consensus was achieved.

Finally, records were independently classified as included, excluded, or requiring further evaluation when ambiguity was identified. Inter-reviewer agreement was assessed using Cohen’s kappa coefficient (κ) [29], revealing a strong level of consistency among reviewers (κ = 0.91). Cases presenting discrepancies were resolved through consensus discussions until a final agreement was reached. This combined iterative and consensus-based approach ensured an appropriate balance between inclusiveness and precision, reducing noise while maintaining comprehensive coverage and methodological robustness throughout the dataset construction process.

### 2.4. Study Selection and PRISMA-Based Filtering Process

**Figure 4:**
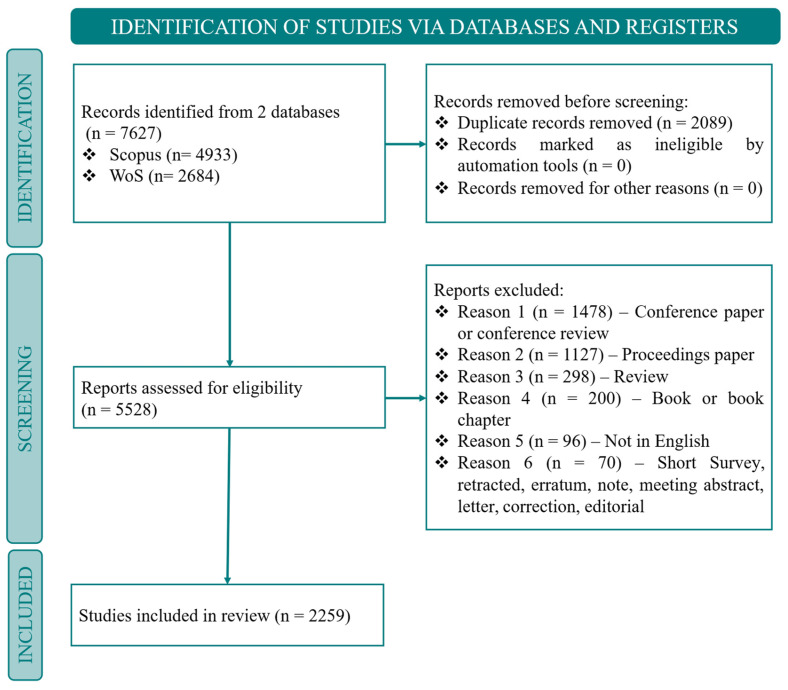
PRISMA flow diagram illustrates the identification, screening, eligibility, and inclusion of studies.

The identification and selection of records followed a structured and transparent workflow aligned with the PRISMA 2020 (Preferred Reporting Items for Systematic Reviews and Meta-Analyses) guidelines, ensuring methodological rigor, reproducibility, and traceability throughout the dataset construction process [30,31]. The PRISMA framework constructed in this work is shown in Figure 4, that enables a systematic approach to screening and filtering this large bibliographic dataset, minimizing selection bias and improving the reliability of the final corpus.

The initial database search yielded a total of 7627 records, including 4933 documents retrieved from Scopus and 2684 from WoS. After merging both datasets, an automated and manual de-duplication process was performed, resulting in the removal of 2089 duplicate records and generating a preliminary dataset of 5528 unique publications for screening.

A multi-stage filtering process was applied to ensure the scientific quality, relevance, and consistency of the dataset, with the objective of retaining only high-quality primary research directly related to the field. In this context, the analysis was restricted to peer-reviewed scientific journal articles written in English, following common practice in bibliometric and science mapping studies, to ensure methodological rigor, source reliability, metadata consistency, and comparability of bibliometric indicators across international research outputs. Consequently, publications that did not correspond to original research articles were excluded, including conference papers and conference reviews (1478), proceedings papers (1127), review articles (298), and books or book chapters (200), to avoid potential biases associated with secondary literature and ensure consistency in bibliometric indicators. Additionally, non-English documents (96) and non-research items—such as editorials, notes, letters, corrections, and short communications (70)—were removed, as they typically lack sufficient methodological and experimental detail for robust bibliometric analysis. Following the full application of inclusion and exclusion criteria, a final dataset of 2259 peer-reviewed research articles was retained for subsequent bibliometric and science mapping analysis.

### 2.5. Data Preprocessing and Science Mapping Techniques

Following the PRISMA-based selection process, the final dataset underwent a preprocessing stage to ensure consistency and analytical reliability. This included the standardization of author names, institutional affiliations, and countries, as well as the normalization of keywords to merge synonymous terms and reduce fragmentation in the analysis. The preprocessing stage involved only limited normalization procedures aimed at correcting minor lexical inconsistencies, including capitalization standardization, singular/plural unification, spelling homogenization, and the merging of exact synonymous abbreviations when unambiguous (e.g., “VR” and “virtual reality”). The analysis combined performance indicators and science mapping techniques. Performance analysis focused on publication output, citation metrics, and the identification of leading authors, institutions, countries, and core journals based on Bradford’s Law.

Science mapping techniques were applied to explore the intellectual and conceptual structure of the field, including co-authorship networks, co-citation analysis, bibliographic coupling, keyword co-occurrence analysis, and thematic evolution analysis. These methods enabled the identification of collaboration patterns, influential research, thematic clusters, and the temporal development of research topics. The analyses were conducted using the Bibliometrix/Biblioshiny framework. Co-occurrence and collaboration networks were constructed from normalized bibliometric matrices using association strength normalization to reduce bias associated with highly frequent terms and strongly connected nodes. For keyword co-occurrence analyses, a minimum occurrence threshold of 15 was established, while collaboration and citation networks were filtered by retaining the strongest 50 connections to improve readability and reduce isolated or weakly connected nodes within the visualizations. Thematic mapping analysis was based on Callon’s centrality and density measures, allowing the classification of themes into motor, basic, niche, and emerging categories according to their relevance and development level within the field. Furthermore, conceptual structure analysis was performed through multiple correspondence analysis (MCA) using authors’ keywords, considering the 50 most frequent terms to identify the principal conceptual dimensions and thematic relationships. Clustering and community detection procedures implemented within Biblioshiny were additionally employed to define thematic groups and network structures using modularity optimization algorithms. Finally, the results were visualized through network graphs and thematic maps generated using Biblioshiny and Origin 2023, ensuring clarity and interpretability of the analyses.

## 3. Results

### 3.1. General Overview of Scientific Production

**Figure 5:**
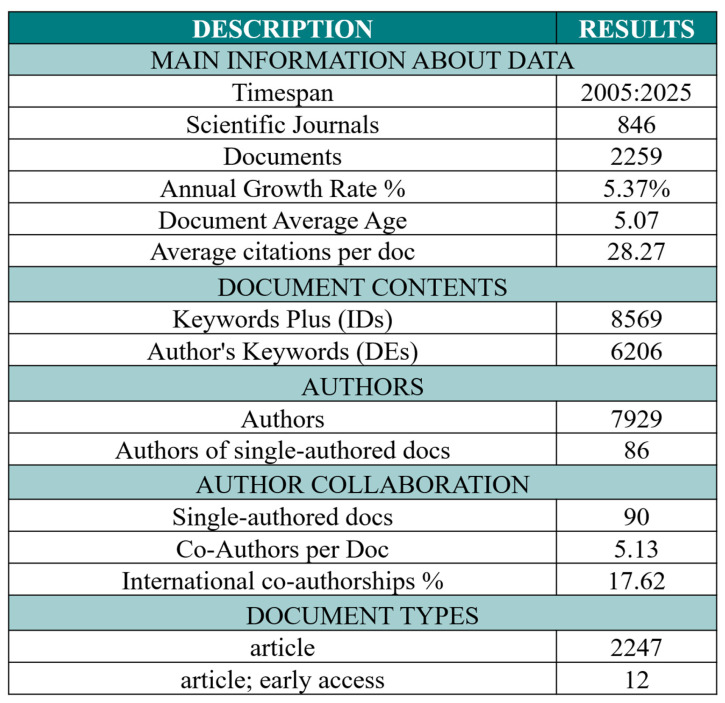
Main bibliometric indicators of the dataset, including timespan, number of documents, scientific journals, authors, keywords, and collaboration metrics.

The general characteristics of the dataset are summarized in Figure 5, which provides a comprehensive overview of the main bibliometric indicators. The analysis covers the period from 2005 to 2025, including a total of 2259 peer-reviewed distributed across 846 scientific journal articles, reflecting the broad and interdisciplinary nature of the research field. The dataset includes contributions from 7929 authors, with a very limited number of single-authored documents (86), highlighting the collaborative nature of research in this domain.

In terms of content, the high number of Keywords Plus (8569) and Authors’ Keywords (6206) indicates a rich and diverse conceptual structure, suggesting that sensing technologies in VR encompass multiple research lines, including materials science, human–computer interaction, biomedical applications, and immersive systems. The average annual growth rate of 5.37% confirms a steady expansion of the field, while the average number of citations per document (28.27) reflects a moderate-to-high scientific impact, consistent with a well-established and actively evolving research area.

Furthermore, the average document age (5.07 years) suggests that a significant portion of the literature is recent, reinforcing the dynamic and emerging character of the topic. Regarding collaboration patterns, the average number of co-authors per document (5.13) indicates a strong tendency toward teamwork and multidisciplinary research. However, the relatively moderate level of international co-authorship (17.62%) suggests that, despite its global relevance, research in this field is still largely structured around national or regional collaboration networks.

**Figure 6:**
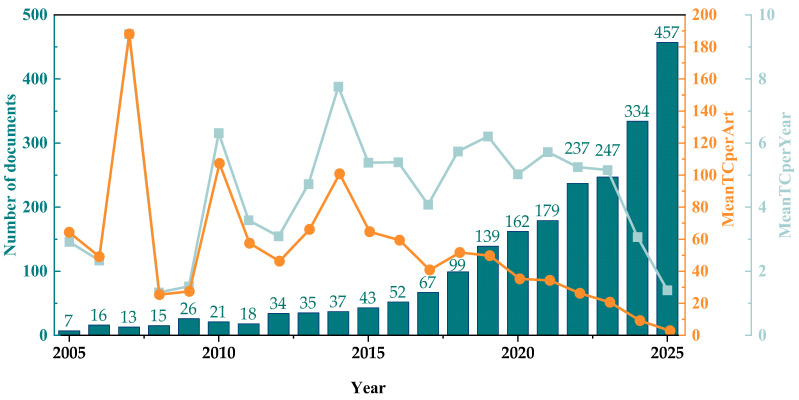
Annual scientific production and citation trends in sensing technologies for VR (2005–2025).

The temporal evolution of scientific production is presented in Figure 6, which illustrates both the annual number of publications and citation trends over time. As observed in Figure 6, the field exhibits three distinct phases of development. The initial phase (2005–2012) is characterized by low and irregular publication output, corresponding to an exploratory stage in which VR technologies and sensing systems were still under development. This is followed by a transition phase (2013–2017), where a gradual increase in publications reflects the growing maturity of VR systems and the incorporation of more advanced sensing technologies. The most significant growth occurs in the consolidation phase (2018–2025), where a sharp increase in the number of publications is observed, reaching a peak of over 450 documents in 2025.

In contrast, citation trends shown in Figure 6 display a decreasing pattern in recent years. This behavior is typical of rapidly expanding research fields, where newly published works have not yet had sufficient time to accumulate citations. Earlier publications, although fewer in number, exhibit higher citation counts, indicating their foundational role in establishing the scientific basis of the field.

### 3.2. Core Scientific Journals and Their Distribution

**Figure 7:**
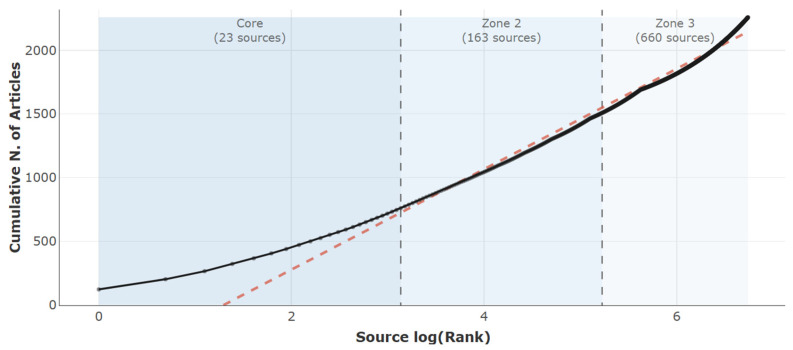
Bradford’s Law distribution of scientific journals, showing the concentration of publications across core, intermediate, and peripheral journal zones. The dashed line corresponds to the linear regression fitted to the dotted-line data.

The distribution of scientific production across scientific journals follows a highly concentrated pattern, as described by Bradford’s Law. This behavior is illustrated in Figure 7, which shows the cumulative number of articles as a function of the logarithmic rank of sources (scientific journals). Here, the literature is clearly divided into three zones: a core zone comprising 23 scientific journals, an intermediate zone with 163 sources, and a peripheral zone including 660 scientific journals. This distribution confirms a strong concentration of publications in a limited number of journals, accompanied by a wide dispersion across secondary scientific journals.

The specific composition of the core scientific journals is detailed in Table 1, which lists the top 10 most productive journals in the field. As shown in Table 1, *Sensors* is the leading journal with 122 publications, representing the main outlet for research on sensing technologies in VR. This is consistent with the technological focus of the field, where sensor development and characterization are central topics. It is followed by *IEEE Transactions on Visualization and Computer Graphics* (80 publications) and *IEEE Access* (63 publications), highlighting the strong contribution of engineering and computer science disciplines.

Other journals within the core zone include *Scientific Reports*, *PLOS ONE*, and *IEEE Transactions on Haptics*, reflecting the multidisciplinary expansion of the field toward experimental, biomedical, and human–machine interaction applications. Additionally, journals such as *Virtual Reality*, *Frontiers in Human Neuroscience*, and *Experimental Brain Research* indicate the increasing integration of immersive technologies with cognitive and neurophysiological research.

The impact indicators reported in Table 2 provide a complementary perspective to the productivity analysis, revealing notable differences between publication volume and scientific influence. Here, the h-index reflects the balance between productivity and citation impact of a journal, the g-index gives greater weight to highly cited publications, and the m-index normalizes the h-index with respect to time, allowing comparison across scientific journals with different publication histories. *IEEE Transactions on Visualization and Computer Graphics* stands out as the most influential journal, with the highest h-index (28) and total citations (3845), despite not being the most prolific scientific journal. In contrast, *Sensors*, although ranking first in terms of number of publications, presents a comparatively lower citation impact, suggesting that it functions primarily as a high-throughput dissemination platform. Moreover, certain journals exhibit high citation efficiency relative to their publication volume. For instance, *Nano Energy* accumulates 1235 citations with only 19 publications, indicating the growing relevance of advanced materials and nanotechnology in VR sensing. Similarly, *Frontiers in Human Neuroscience* and *Virtual Reality* show strong citation performance, emphasizing the importance of neurophysiological and immersive interaction studies.

### 3.3. Institutions, Countries, and Global Research Distribution

The analysis of institutional productivity (Table 3) reveals a strong concentration of research activity within leading international institutions, with a clear dominance of organizations from the United States and China. Pennsylvania State University ranks as the most productive institution with 138 publications, followed by the Chinese Academy of Sciences (67 publications), the University of California (65 publications), and the National University of Singapore (60 publications). This distribution highlights the strategic role of large and well-funded research institutions in driving advancements in VR sensing technologies and immersive interaction systems. Several institutions from the United States (including Pennsylvania State University, the University of California, the University of Washington, and Stanford University) appear prominently, confirming the strong influence of North American research ecosystems within the field. At the same time, the presence of institutions such as Zhejiang University, Tsinghua University, the University of London, and the Swiss Federal Institutes of Technology reflects the highly international and competitive nature of research in VR sensing technologies.

At the country level, the distribution of scientific production (Table 4) further reinforces these trends. China (407 publications, 18.0%) and the United States (401 publications, 17.8%) clearly dominate the field, followed by South Korea, Italy, and the United Kingdom. This concentration indicates that research in VR sensing is largely driven by technologically advanced economies with strong investments in digital and immersive technologies. The analysis of collaboration patterns reveals a moderate degree of international cooperation. While most publications are produced within single-country frameworks (SCP), a notable proportion of multi-country collaborations (MCP) is observed, particularly in countries such as China, the United Kingdom, and Italy. However, the overall level of international co-authorship suggests that research efforts remain partially fragmented across national boundaries.

**Figure 8:**
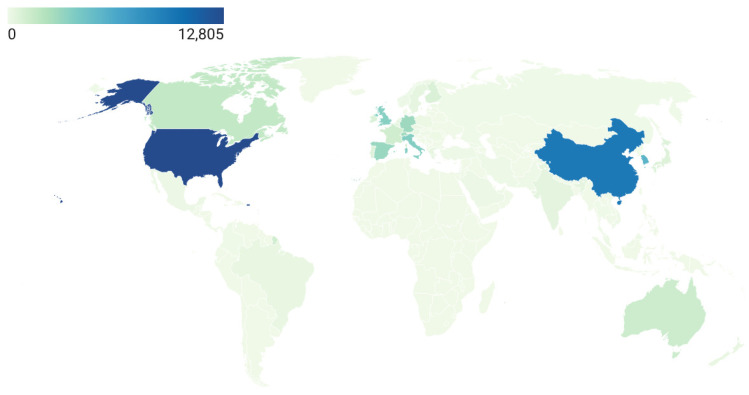
Global distribution of citations and average citation impact by country in VR sensing research.

The global distribution of scientific impact is illustrated in Figure 8, which maps total citations and average citation impact by country. As shown in Figure 8, the United States leads in total citations (12,805), followed by China (9619) and South Korea (5674), reflecting both high productivity and strong research influence. However, when considering average citations per article, smaller research systems such as Finland (131), Singapore (78.9), and Switzerland (56.9) exhibit significantly higher impact, suggesting the presence of highly specialized and influential contributions.

### 3.4. Most Influential Publications

The most cited documents in the field are summarized in Table 5, providing an overview of the key publications that have significantly contributed to the development of sensing technologies in VR. These works span different domains, including materials science, human perception, and immersive interaction, reflecting the multidisciplinary nature of the field. In addition to total citations, indicators such as citations per year and normalized citation impact allow the identification of both foundational studies and more recent high-impact contributions.

## 4. Discussion

### 4.1. Global Evolution and Consolidation of VR Sensing Research

The present bibliometric analysis reveals a rapidly evolving and increasingly structured research landscape in sensing technologies for VR, characterized by strong interdisciplinarity and technological convergence. The sustained growth in scientific production, together with the high collaboration rates observed across authors, institutions, and countries, reflects the progressive consolidation of the field from an emerging research area toward a more mature and diversified scientific domain. Here, the concentration of publications within a limited number of core journals, combined with the broad distribution across multidisciplinary scientific journals, highlights both the existence of established dissemination platforms and the inherently interdisciplinary character of VR sensing research, integrating engineering, computer science, materials science, neuroscience, and human–computer interaction. From a global perspective, the bibliometric results additionally reveal a dual research structure, where high-output countries dominate in terms of publication volume, while other countries achieve comparatively higher citation efficiency despite lower scientific production. This tendency may reflect differences in research strategies, funding capacities, and specialization levels across national research systems. Consequently, the combined analysis of institutions, countries, journals, and citation impact highlights a geographically concentrated yet globally interconnected scientific ecosystem, in which leading economies drive scientific production while smaller and more specialized systems contribute disproportionately to high-impact research.

### 4.2. Major Research Pillars Based on Influential Publications

Among the most highly cited publications identified in Table 5, several dominant thematic directions can be observed, reflecting the interdisciplinary nature of VR sensing research and its progressive evolution toward human-centered immersive systems. Here, three major research pillars can be distinguished: (i) advanced sensing materials and wearable technologies, (ii) human perception and multisensory integration, and (iii) applications of VR in interaction, training, and intelligent systems.

The first major research pillar identified among the most influential publications is associated with advanced sensing materials and wearable technologies applied to human motion monitoring and immersive interaction. Highly cited works focused on highly stretchable strain sensors based on nanocomposite materials, including silver nanowires and graphene-elastomer systems, demonstrated the growing relevance of flexible sensing technologies for real-time human motion detection [32,35]. More recent contributions involving triboelectric smart gloves and deep learning-enabled smart socks further illustrate the increasing convergence between wearable sensing, artificial intelligence, Internet-of-Things architectures, and immersive VR applications [38,40].

A second highly influential thematic pillar is centered on human perception, embodiment, and multisensory integration. Foundational studies investigating bodily self-consciousness, full-body ownership illusion, and multisensory integration mechanisms demonstrated how sensorimotor feedback and immersive interaction modulate human perception and embodiment within VR environments [33,36,41]. These works reveal that VR interaction extends beyond purely hardware-oriented sensing systems and increasingly incorporates cognitive, perceptual, and neurophysiological dimensions related to user experience.

The third major research direction involves the application of VR technologies to immersive interaction, navigation, memory enhancement, training, and intelligent environments. Influential studies on redirected walking techniques significantly contributed to the development of spatial interaction and locomotion strategies in immersive VR systems [34], while other works demonstrated the potential of immersive environments for improving memory recall and cognitive performance [37]. In parallel, immersive VR applications in cultural heritage and museum environments further illustrate the broad applicability of sensing-enabled VR systems beyond traditional engineering contexts [39].

In view of these thematic pillars, the coexistence of engineering-oriented sensing studies with embodiment, perception, and application-driven VR research further reinforces the broad conceptual framework adopted throughout the present bibliometric analysis and helps explain the interdisciplinary thematic structures identified within the science mapping results.

### 4.3. Conceptual Structure

**Figure 9:**
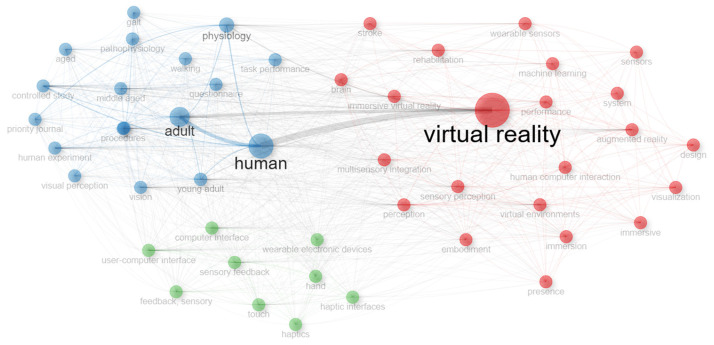
Keyword co-occurrence network revealing the conceptual structure of sensing technologies in VR for human interaction.

The keyword co-occurrence network (Figure 9) highlights *virtual reality* as the central node around which the entire conceptual structure is organized, strongly connected with terms such as *human*, *sensory perception*, *immersion*, and *human–computer interaction*. This topology reflects a clear transition from technology-driven research toward interaction-driven paradigms, where the user is placed at the core of system design. In this context, sensing technologies act as the fundamental interface between physical and virtual environments, enabling real-time capture and interpretation of user actions and states [42,43]. The presence of clusters related to *wearable sensors*, *haptics*, and *feedback systems* further indicates the growing importance of embodied interaction, where the body itself becomes a key component of the interface [44].

This shift is consistent with broader technological trends emphasizing natural interaction and multisensory integration, where visual immersion alone is no longer sufficient. Instead, the field is moving toward systems capable of capturing mechanical, physiological, and cognitive signals simultaneously [45]. This is particularly relevant for applications in healthcare, rehabilitation, and training, where accurate sensing of human responses is essential.

### 4.4. Thematic Structure

**Figure 10:**
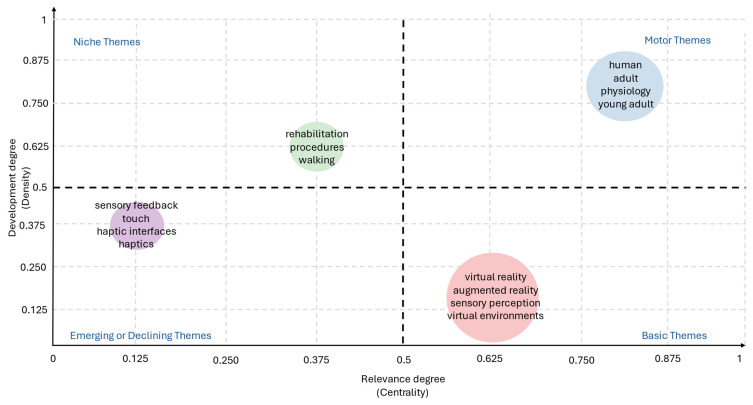
Thematic map based on co-word analysis showing the distribution of research topics according to centrality and density (motor, basic, niche, and emerging themes).

The thematic map (Figure 10) provides further insight into the maturity and strategic importance of different research topics. Core themes such as *virtual reality*, *augmented reality*, and *sensory perception* are positioned as basic themes, indicating that they form the foundational knowledge base of the field. In contrast, *human physiology*, *adult*, and related terms appear as motor themes, characterized by both high centrality and density, suggesting that physiological sensing and human-centered analysis are currently driving the evolution of VR research.

However, one of the most relevant findings is the positioning of *haptics*, *touch*, and *sensory feedback* within the emerging or weakly developed quadrant. This indicates that, despite their recognized importance for achieving full immersion, these topics appear comparatively less represented within the analyzed bibliometric thematic structures. This thematic positioning suggests potential opportunities for future research related to haptic interaction and sensory feedback within immersive VR environments. The limited maturity of these themes suggests that future research should prioritize the development of robust, scalable, and high-fidelity tactile sensing and feedback systems [46]. This interpretation is additionally supported by the comparatively lower frequency, centrality, density, and connectivity associated with haptics, touch, and sensory feedback-related keywords within the co-occurrence and thematic network structures.

Similarly, niche themes such as *rehabilitation*, *walking*, and *procedures* reflect specialized application domains that, although not central to the overall structure, exhibit high development potential. These areas are likely to grow as VR becomes more widely adopted in clinical and training environments [47].

### 4.5. Factorial Analysis and Conceptual Dimensions

**Figure 11:**
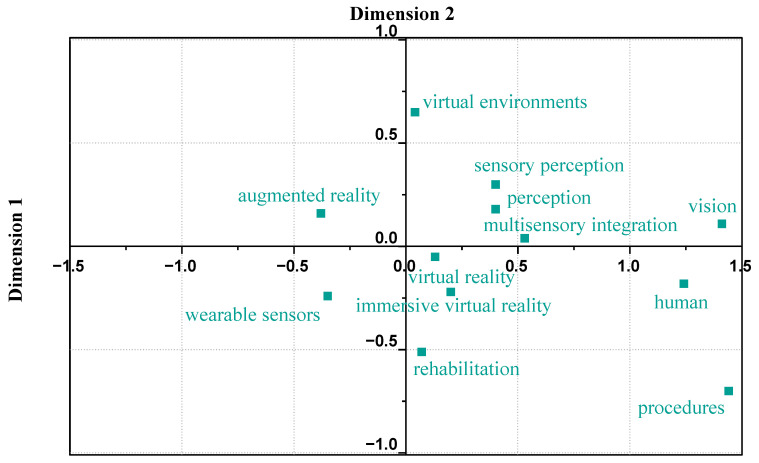
Conceptual structure map based on factorial analysis illustrating the relationships and dimensions of key research themes.

The factorial analysis (Figure 11) further elucidates the conceptual structure of the field by revealing two dominant dimensions. On one side, keywords associated with the technological and system-oriented dimension—such as *augmented reality* (−0.38, 0.16), *virtual environments* (0.04, 0.65), and *wearable sensors* (−0.35, −0.24)—are positioned toward the left-hand side of the map, reflecting their role in defining the infrastructural and hardware-related aspects of VR systems. On the opposite side, terms such as *human* (1.24, −0.18), *adult* (1.67, −0.28), *physiology* (1.74, 0.28), and *young adult* (2.07, 0.30) are clearly shifted toward the positive side of Dimension 1, indicating a strong alignment with human-centered and physiological research approaches.

Additionally, interaction-related terms such as *user–computer interface* (2.03, 0.69) and *computer interface* (2.08, 0.89) show high positive values in both dimensions, suggesting their central role in bridging technological systems and user interaction. In contrast, perception-related keywords such as *sensory perception* (0.40, 0.30), *perception* (0.40, 0.18), and *multisensory integration* (0.53, 0.04) occupy intermediate positions, reinforcing their function as connecting elements between system-level and human-level processes.

Particularly noteworthy is the positioning of *sensory feedback* (1.68, 2.43) and *touch* (1.22, 2.18), which exhibit high values along Dimension 2, indicating strong development but relatively limited integration within the broader conceptual structure. This reinforces the idea that haptic and tactile interaction, despite being technically advanced, remain insufficiently connected to the core VR research framework. Similarly, terms such as *rehabilitation* (0.07, −0.51) and *procedures* (1.44, −0.70) appear in more peripheral positions, reflecting their application-specific nature.

This distribution highlights a persistent separation between technological development and human-centered evaluation, with only a limited number of concepts effectively bridging both dimensions. This conceptual distribution may indicate future research directions associated with the integration of wearable and bio-integrated sensing approaches within VR interaction research.

### 4.6. International Collaboration and Global Research Ecosystem

**Figure 12:**
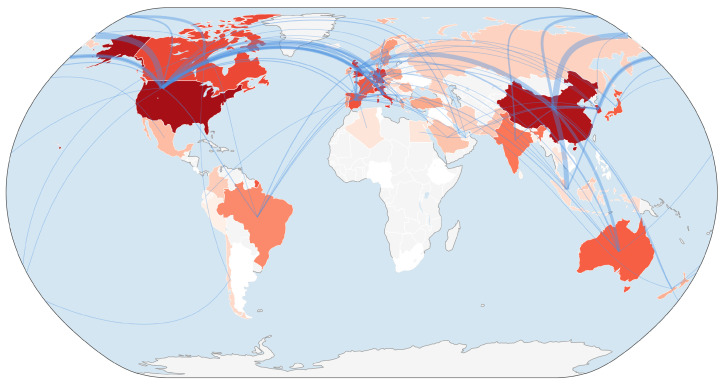
Global collaboration network of countries in VR sensing research, highlighting international research connections and geographical distribution.

From a global perspective, the collaboration network (Figure 12) reveals a geographically concentrated yet interconnected research landscape. Major hubs such as the United States and China dominate in terms of both production and connectivity, forming the backbone of the global research network. At the same time, strong collaboration links with European countries indicate the presence of well-established international partnerships. Nevertheless, the network also reveals a degree of fragmentation, with several regions exhibiting limited integration into the global research ecosystem. This suggests that, despite increasing international collaboration, there is still room for improving knowledge exchange and cross-regional cooperation, particularly in emerging research areas.

Another relevant aspect is the distinction between high-output and high-impact research systems. While large countries dominate in terms of publication volume, smaller systems often achieve higher citation impact, indicating greater specialization and efficiency. This pattern suggests that future progress in the field will not depend solely on research volume but also on the ability to generate high-quality, innovative, and interdisciplinary contributions.

### 4.7. Future Trends, Challenges and Limitations

Taken together, all the results point toward several key trends shaping the future of sensing technologies in VR. First, there is a clear move toward multimodal sensing, integrating mechanical, optical, and physiological signals to achieve more comprehensive interaction models. Second, the increasing importance of bio-integrated sensing reflects the need to incorporate cognitive and emotional dimensions into VR experiences. Third, the development of flexible and nanomaterial-based sensors is expected to play a critical role in enabling next-generation wearable systems.

Despite these advances, several challenges remain. These include the integration of heterogeneous sensing technologies, the long-term stability and reliability of wearable sensors, and the lack of standardized evaluation protocols for sensor performance in VR environments. Additionally, issues related to scalability, power consumption, and data processing must be addressed to enable the widespread adoption of advanced sensing systems. In this context, nanotechnology-based sensors, particularly those based on carbon nanotubes, graphene, and hybrid nanocomposites, offer promising pathways due to their high sensitivity, flexibility, and compatibility with wearable platforms. However, their practical implementation is still limited by challenges such as signal drift, hysteresis effects, large-scale manufacturability, and long-term durability under real operating conditions [48,49,50].

Despite the robustness and comprehensive nature of the bibliometric framework adopted in this study, several methodological limitations should be acknowledged. (i) The analysis was restricted to the Scopus and Web of Science databases, which, although recognized as highly standardized and comprehensive scientific journals for bibliometric studies, may not fully capture publications indexed exclusively in other databases or regional repositories. (ii) Only peer-reviewed scientific journal articles were included in the final dataset to ensure methodological rigor, metadata consistency, and comparability of bibliometric indicators. However, conference proceedings and conference papers could constitute important dissemination channels for emerging technologies and early-stage developments. Consequently, some recent or highly specialized research trends may be underrepresented in the present analysis. (iii) The bibliometric methodology is inherently dependent on the selected search strategy, keyword structure, and preprocessing procedures. Therefore, despite the iterative refinement, pilot searches, and normalization processes employed throughout the study, some relevant publications may not have been retrieved due to differences in terminology, indexing practices, or database-specific classifications. (iv) Average citation indicators associated with smaller research systems should be interpreted cautiously, as a limited number of highly cited publications may disproportionately affect them.

**Figure 13:**
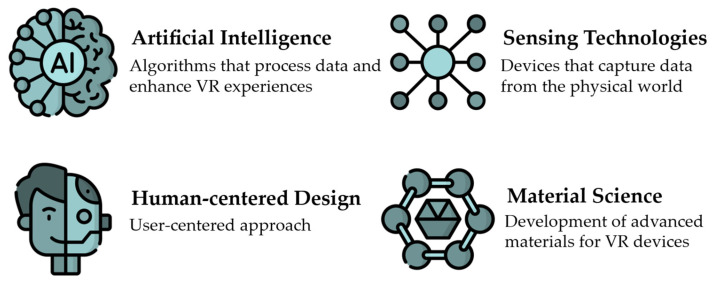
Future directions for integrated VR sensing systems.

To sum up (Figure 13), the field is transitioning toward a new paradigm characterized by the convergence of sensing, materials science, artificial intelligence, and human-centered design. In this context, future research efforts should focus on developing integrated, adaptive, and intelligent sensing platforms capable of bridging the gap between physical and virtual worlds, ultimately enabling more immersive, responsive, and personalized VR experiences.

## 5. Conclusions

This study provides a comprehensive bibliometric and science mapping analysis of sensing technologies in virtual reality for human interaction, offering a structured and quantitative overview of a rapidly evolving and highly interdisciplinary research field. The results demonstrate a sustained growth in scientific production, with 2259 peer-reviewed articles published between 2005 and 2025 across 846 scientific journals, and an average annual growth rate of 5.37%.

The analysis reveals a well-defined scientific structure characterized by a strong concentration of publications within a limited number of core journals, with 23 scientific journals forming the core zone according to Bradford’s Law, alongside a broader dispersion across 660 peripheral scientific journals. At the same time, the distinction between high-productivity and high-impact journals highlights the dual dynamics of knowledge dissemination and intellectual influence, where leading outlets combine high publication output with strong citation performance.

From a geographical perspective, research activity is dominated by major scientific hubs such as China (18.0% of total publications) and the United States (17.8%), which also lead in total citations (12,805 and 9619, respectively). However, smaller research systems exhibit higher citation efficiency, with countries such as Finland reaching an average of 131 citations per article, indicating the importance of specialized and high-impact contributions within the global research ecosystem.

From a conceptual standpoint, the bibliometric trends suggest an increasing research interest toward human-centered and interaction-driven approaches, where sensing technologies play a central role in enabling realistic and adaptive virtual environments. The thematic structures and keyword co-occurrence analyses suggest an increasing research interest in multimodal and bio-integrated sensing approaches involving mechanical, optical, and physiological sensing technologies associated with immersive human interaction. However, comparatively lower thematic representation remains observable, particularly in the development and integration of haptic and tactile feedback systems, which continue to represent a potential limitation or research challenge for achieving more immersive VR experiences. Furthermore, the increasing presence of flexible, wearable, and nanomaterial-based sensors within the bibliometric structures reflects the growing thematic relevance of materials science within VR sensing research.

Future research may further explore the integration of sensing technologies, advanced materials, artificial intelligence, and human-centered interaction approaches within immersive VR environments.

## Figures and Tables

**Table 1:** Top 10 most productive journals in sensing technologies for VR.

Scientific Journal	Rank	Frequency	Cumulative Frequency	Zone
Sensors	1	122	122	Zone 1
IEEE Transactions on Visualization and Computer Graphics	2	80	202	Zone 1
IEEE Access	3	63	265	Zone 1
Scientific Reports	4	57	322	Zone 1
PLOS ONE	5	44	366	Zone 1
IEEE Transactions on Haptics	6	39	405	Zone 1
Virtual Reality	7	34	439	Zone 1
Frontiers in Human Neuroscience	8	32	471	Zone 1
Frontiers in Virtual Reality	9	28	499	Zone 1
Experimental Brain Research	10	26	525	Zone 1

**Table 2:** Citation impact indicators of leading journals in VR sensing research.

Scientific Journal	h-Index	g-Index	m-Index	Total Citations	Number of Publications
IEEE Transactions on Visualization and Computer Graphics	28	61	1.273	3845	80
Sensors	24	37	2.182	1869	122
Scientific Reports	21	38	1.615	1546	57
PLOS ONE	19	40	1.267	1670	44
Nano Energy	16	19	2.000	1235	19
Frontiers in Human Neuroscience	15	32	1.071	1244	32
Virtual Reality	15	34	0.714	1918	34
Experimental Brain Research	14	26	0.700	735	26
IEEE Transactions on Haptics	14	31	0.875	1009	39
Journal of NeuroEngineering and Rehabilitation	13	26	0.650	811	26

**Table 3:** Leading institutions and their country distribution in VR sensing research.

Affiliation	Country	Number of Articles
Pennsylvania State University	United States	138
Chinese Academy of Sciences	China	67
University of California	United States	65
National University of Singapore	Singapore	60
University of London	United Kingdom	47
Zhejiang University	China	42
Swiss Federal Institutes of Technology	Switzerland	39
University of Washington	United States	33
Stanford University	United States	32
Tsinghua University	China	24

**Table 4:** Corresponding authors’ countries, including single-country (SCP) and multi-country publications (MCP).

Country	Number of Articles	Articles (%)	SCP	MCP
China	407	18.0	314	93
United States	401	17.8	354	47
South Korea	163	7.2	147	16
Italy	154	6.8	128	26
United Kingdom	121	5.4	93	28
Germany	112	5.0	91	21
Spain	76	3.4	59	17
Canada	73	3.2	64	9
France	65	2.9	58	7
Japan	57	2.5	52	5

**Table 5:** Top 10 most cited papers in sensing technologies for VR, including total citations, citations per year, and normalized citation impact.

Ref	Paper (First Author, Year, Journal)	Total Citations	Citations Per Year	Normalized TC
[32]	Amjadi M. (2014), ACS Nano	2257	173.6	22.38
[33]	Lenggenhager B. (2007), Science	1030	51.5	5.48
[34]	Steinicke F. (2010), IEEE Trans. Vis. Comput. Graph.	594	34.9	5.54
[35]	Park J. (2015), ACS Appl. Mater. Interfaces	539	44.9	8.33
[36]	Maselli A. (2013), Front. Hum. Neurosci.	501	35.8	7.58
[37]	Krokos E. (2019), Virtual Reality	470	58.8	9.46
[38]	Wen F. (2021), Nature Communications	447	74.5	13.02
[39]	Carrozzino M. (2010), J. Cult. Herit.	394	23.2	3.67
[40]	Zhang Z. (2020), npj Flex. Electron.	385	55.0	10.92
[41]	Suzuki K. (2013), Neuropsychologia	383	27.4	5.80

## Data Availability

The original contributions presented in this study are included in the article; further inquiries can be directed to the corresponding author.
